# Evaluating semantic similarity methods for comparison of text-derived phenotype profiles

**DOI:** 10.1186/s12911-022-01770-4

**Published:** 2022-02-05

**Authors:** Luke T. Slater, Sophie Russell, Silver Makepeace, Alexander Carberry, Andreas Karwath, John A. Williams, Hilary Fanning, Simon Ball, Robert Hoehndorf, Georgios V. Gkoutos

**Affiliations:** 1 College of Medical and Dental Sciences, Institute of Cancer and Genomic Sciences, University of Birmingham, Birmingham, UK; 2 Institute of Translational Medicine, University Hospitals Birmingham, NHS Foundation Trust, Birmingham, UK; 3 NIHR Experimental Cancer Medicine Centre, Birmingham, UK; 4 NIHR Surgical Reconstruction and Microbiology Research Centre, Birmingham, UK; 5 NIHR Biomedical Research Centre, Birmingham, UK; 6 MRC Health Data Research UK (HDR UK) Midlands, Birmingham, UK; 7 Computational Bioscience Research Center, Computer, Electrical and Mathematical Sciences and Engineering Division, King Abdullah University of Science and Technology, Thuwal, Saudi Arabia; 8 University Hospitals Birmingham NHS Foundation Trust, Edgbaston, Birmingham, UK

**Keywords:** Semantic web, Ontology, Differential diagnosis, MIMIC-III, Semantic similarity

## Abstract

**Background:**

Semantic similarity is a valuable tool for analysis in biomedicine. When applied to phenotype profiles derived from clinical text, they have the capacity to enable and enhance ‘patient-like me’ analyses, automated coding, differential diagnosis, and outcome prediction. While a large body of work exists exploring the use of semantic similarity for multiple tasks, including protein interaction prediction, and rare disease differential diagnosis, there is less work exploring comparison of patient phenotype profiles for clinical tasks. Moreover, there are no experimental explorations of optimal parameters or better methods in the area.

**Methods:**

We develop a platform for reproducible benchmarking and comparison of experimental conditions for patient phentoype similarity. Using the platform, we evaluate the task of ranking shared primary diagnosis from uncurated phenotype profiles derived from all text narrative associated with admissions in the medical information mart for intensive care (MIMIC-III).

**Results:**

300 semantic similarity configurations were evaluated, as well as one embedding-based approach. On average, measures that did not make use of an external information content measure performed slightly better, however the best-performing configurations when measured by area under receiver operating characteristic curve and Top Ten Accuracy used term-specificity and annotation-frequency measures.

**Conclusion:**

We identified and interpreted the performance of a large number of semantic similarity configurations for the task of classifying diagnosis from text-derived phenotype profiles in one setting. We also provided a basis for further research on other settings and related tasks in the area.

## Background

Analysis of natural language in clinical settings facilitates insight into relevant biomedical entities, which in turn can lead to improved outcomes for patients [1, 2]. Biomedical ontologies are frequently employed as resources in natural language processing (NLP) analyses, aiding in the resolution and reduction of ambiguity through their provision of controlled domain vocabularies and consensus definitions of biomedical concepts [3].

Moreover, linking instances of biomedical concepts mentioned in text with ontology classes produces semantic representations of entities described by those texts. These representations facilitate secondary analyses that make use of background knowledge encoded in or linked by ontologies. One such method for semantic analysis is semantic similarity: a class of methods that leverage the structural features of ontologies to calculate numerical measures of similarity between classes or sets of classes [4, 5]. These methods have been widely explored, amongst others, for prediction of protein–protein interaction [6], disease gene prioritisation [7, 8], and rare disease diagnosis [9].

While semantic similarity has been widely explored for many tasks in biomedicine, it has only more recently been applied to phenotype profiles text-mined from clinical narrative text. One work used similarity of phenotypes text-mined from literature to characterise the human diseasome [10]. Another investigation developed Doc2HPO, which uses a hybrid approach of concept recognition with expert curation to produce phenotype profiles that can be analysed using several variant prioritisation methods [11]. Another work explored the use of uncurated text-derived phenotypes for differential diagnosis of common diseases [12].

Benchmarking tasks have been defined for the comparison of semantic similarity measures in several biomedical spaces. For example, the collaborative evaluation of semantic similarity measures (CESSM) task supports collaborative comparison of semantic similarity approaches for tasks involving the gene ontology (GO) [13]. In the clinical space, experiments were performed to evaluate effectiveness at measuring similarity between classes in medical terminologies [14, 15]. To our knowledge, however, there are no such evaluations pertaining to predictive clinical tasks using patient phenotype profiles, despite this being an emerging area of application for semantic similarity. Nor are we aware of any evaluations for clinical semantic similarity tasks using text-derived phenotype profiles. There is, therefore, a lack of knowledge surrounding best practices and configurations for this area of problems. Implementation of comparisons are also difficult, since there are no well-defined tasks or frameworks that can be easily used for benchmarking, or infrastructure surrounding these uses, even though such approaches have proven useful in other areas (e.g. with Gene Ontology).

Previous work comparing semantic similarity methods on a single hierarchical medical terminology, SNOMED-CT [16], revealed poor agreement between methods [15]. Other works comparing semantic similarity methods for different tasks using GO found that different methods were more suitable for different tasks [5]. Differences in performance may also arise between different ontologies due to differing design principles, structure, breadth, or complexity.

Furthermore, the use of semantic similarity on large text-derived phenotype profiles is challenging. In one recent work, concept recognition performed on clinical narrative associated with 1000 medical information mart for intensive care (MIMIC-III) admissions with the human phenotype ontology (HPO) [17] producing 43,953 separate annotations, with a mean of nearly 44 annotations per admission [18]. Other work has shown that greater annotation size leads to bias in semantic similarity calculations, in the form of greater similarity between entities with more annotations, regardless of whether they are more biologically related [19]. These findings reveal the need for investigations comparing semantic similarity methods for clinical tasks using phenotype profiles, and for clinical tasks using uncurated text-derived phenotype profiles. Direct clinical uses for these methods include variant prioritisation, patient stratification, and differential diagnosis for rare and common diseases; thus knowledge surrounding the best configurations may inform improvements to developments or implementations of these systems. For example, similarity-driven approaches from Monarch [20], PhenomeNET [8], Phenodigm [21], and Exomiser [22] have been used to prioritize variants in patients with rare disease. Importantly, using semantic similarity for clinical tasks or to characterise population health has real world consequences and direct translational impact not directly seen in other semantic similarity tasks such as protein–protein interaction or gene function prediction. Because of the importance of evaluating positive and negative predictive value in clinical cases, a reproducible, patient-focused benchmark of similarity measures is acutely needed.

Our work contributes to these proposed explorations by establishing a platform for evaluating relevant tasks, and by performing, and presenting the results of, one such exploration. In this work, we describe the development of a platform for reproducible and repeatable evaluation of different experimental conditions for comparison of patient phenotype profiles. We use this platform to compare and report upon performance of different semantic similarity methods for predicting shared primary patient diagnosis using uncurated text-derived phenotype profiles, and report upon the results of this investigation, identifying best-performing methodological configurations. We anticipate that our results and platform will inform future approaches employing semantic similarity across a number of tasks including patient/disease stratification, multimorbidity analysis and clustering, pathogenic variant prioritisation, and differential disease diagnosis.

## Semantic similarity

Semantic similarity measures compute a quantitative heuristic of similarity between multiple concepts. Determining similarity between concepts is useful for many biomedical and non-biomedical tasks, and can be constructed as an unsupervised machine learning problem. Similarity can be calculated between words and sentences in natural text [23], nodes in graphs, or through logical relations in ontologies or related constructs [5]. All measures, regardless of knowledge domain, require a particular representation that can be computed; while many modes of semantic similarity can be more loosely defined upon graphs or other methods, we will focus on ontology-based methods.

Similarity can be computed for classes, annotated entities, or individuals in biomedical ontologies, and these measures are most often defined through some consideration of the ontology structure as a directed acyclic graph, most frequently representing the subsumptive taxonomy of the ontology once classified by a reasoner [4]. Ontology graphs are composed of classes of entities (e.g., a phenotype) and relational edges between entities. We can represent this by $A \sqsubseteq B$, where class B is a more specific subclass of class A. This expression assumes a subsumptive *is_a* relation. While other relations often exist, ranging from *part_of* and *temporally_related_to*, *is_a* relations and their enforced subsumptive nature are exclusively used by many semantic similarity measures.

Pairwise similarity methods calculate similarity between a single pair of terms in a biomedical ontology. For example, the $\hbox {Sim}_{{Rada}}$ measure is defined in terms of the shortest path between two given nodes in a an ontology graph:

1 $$\begin{aligned} Sim_{Rada}(x,y) = \frac{1}{dist_{SP}(x,y)+1} \end{aligned}$$

where $dist_{SP}$ is the shortest path between two nodes in the the graph [24]. This measure, and others like it, rely on the structure of the graph alone and do not take into account the relational nature of ontological structure: if the distance between two classes is similar to that expected of two arbitrary unrelated classes, this will not be captured. Information content measures can partially mitigate this problem. An information content measure is a method that determines how specific or informative a particular term is. These can also be structural, such as in the case of the Zhou et al. [25] method, which uses a measure of how deep a term is in the ontology graph, or corpus-based, such as the Resnik [26] method, which uses a measure of how likely a term is to appear in a corpus. This is simply defined as:

2 $$\begin{aligned} IC_{Resnik}(x) = -\log p(x), \end{aligned}$$

with

3 $$\begin{aligned} p(x) = \frac{\left| I(x) \right| }{\left| I\right| } \end{aligned}$$

where *I* is the full set of annotations (e.g. patient-phenotype associations) and *I*(*x*) is the subset of annotations linked to *x* or its subclasses. Each pairwise similarity method that admits an information content measure will use it in a different way, such as being defined as the information content of the most common ancestor of the two input classes. A prime example is again from Resnik [26], where:

4 $$\begin{aligned} Sim_{Resnik}(x,y) = IC(MICA(x,y)) \end{aligned}$$

where *MICA* is the most informative common ancestor of both *x* and *y*.

Phenotype profiles, however, generally contain more than one phenotype to describe an entity. To calculate semantic similarity between two sets of ontology classes, groupwise measures can be employed. These are further delineated into two groups. Direct groupwise measures define their own methods of directly calculating similarity between two sets of classes. Indirect groupwise measures, however, rely on a pairwise method given as a parameter, employing it as a constituent of the method. For example, the $Sim_{max}$ indirect groupwise measure compares each term in the two input sets using a given pairwise similarity method, and then selects the greatest score as the result:

5 $$\begin{aligned} Sim_{max}(A,B) = MAX_{1 \le i \le m,1 \le j \le n} Sim(A_i, B_j) \end{aligned}$$

A common groupwise approach not relying on pairwise similarity is the Jaccard index between the sets of entities to which patients may be annotated:

6 $$\begin{aligned} Jaccard(A, B) = \frac{|A \bigcap B|}{ |A\bigcup B|} \end{aligned}$$

which relies solely on the annotation corpus.

Recent developments in semantic similarity have taken advantage of diverse graph substructures via syntactic embeddings and representation learning. In DeepWalk, relations between entities within ontology graphs can be explored by random walks, in which walks of a given length are taken between nodes/classes in the graph to generate “sentences” [27]. Embeddings are then created from these sentences, via Word2Vec, hearkening back to the beginnings of semantic similarity as applied to a corpus of written text [28]. The correlation of these embeddings then represents the similarity of entities. Extensions of these approaches have incorporated ontology-specific structure itself [29], effectively deriving new measures of semantic similarity. However, the non-deterministic nature of these embeddings and the ubiquity of more formalized measures of semantic similarity leave us to focus on traditional methods in wider use.

## Methods

**Fig. 1:**
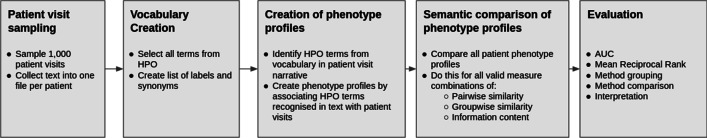
Overall description of the experimental methodology. These processes are split into separate notebooks, forming modules that can be modified and replaced, to extend the framework to explore other methods, outcomes, or settings

We developed a framework with which to perform reproducible semantic similarity experiments using HPO-based phenotype profiles, using the MIMIC-III dataset [30]. While the software can be used to perform experiments with phenotype profiles of any etiology, for the purposes of our investigation we developed a workflow that creates uncurated text-derived phenotype profiles for evaluation. The modular nature of the implementation allows for alternative means of producing or importing phenotype profiles or running similar experiments, making it possible to use it as a basis for exploration of other outcomes, datasets, modalities, or related tasks. The platform is implemented in the form of Jupyter Notebooks containing executable code for repeating, modifying, and evaluating the experiments. The software is available, with instructions, from https://github.com/reality/mimpred. The overall experimental methodology, visualised in Fig. 1, centres around sampling admissions, obtaining the associated clinical narrative text, producing phenotype profiles, then calculating and evaluating a large set of semantic similarity methods using a common interface. The subsequent methods subsections describe these steps in detail.

### Sampling admissions

Our evaluation employed the MIMIC-III database [30]. MIMIC is a freely available database of healthcare data describing nearly 60,000 visits to a critical care clinic at Beth Israel Deaconess Medical Center in Boston, Massachusetts. It provides a wealth of structured and unstructured data concerning those visits, including clinical narrative text. Diagnoses for patients are also provided in the form of ICD-9 codes, which were produced following visits by professional curators.

We sampled 1000 admissions, each describing a single patient visit, from the MIMIC dataset, collecting their associated texts together into one file per patient visit. Texts fall under different categories, such as discharge notes, nursing notes, and test results. In this experiment, we concatenate all notes from all categories, to provide a baseline. Future work could consider comparing different groups of note categories to evaluate any potential effect on performance. We also performed pre-processing to collapse multiple line breaks into sentence breaks, and otherwise to improve formatting of the files. Finally, we associated each admission with its primary diagnosis given by structured coding data.

### Vocabulary creation and creation of phenotype profiles

We then used the Komenti semantic text mining framework [31] to create a vocabulary from all non-obsolete terms in the Human Phenotype Ontology (HPO), which describes phenotypic abnormalities in humans [17]. Komenti was then applied annotate the texts, identifying HPO terms from the vocabulary in the clinical narrative associated with each admission. The set of HPO terms identified with each admission constitutes that the phenotype profile for that admission.

### Semantic comparison of phenotype profiles and evaluation

We subsequently created sets of similarity scores for each pairwise combination of phenotype profiles derived from the text associated with the 1000 sampled admissions. To calculate the semantic similarity scores, we used the Semantic Measures Library toolkit (SML) [32], and we explored every available combination of information content, pairwise, and groupwise similarity measure available within the library. The SML toolkit was chosen because it includes a large range of reference implementations for rule-based semantic similarity measures. Moreover, it provides a common interface for those measures under a single abstraction, ensuring that results are fully comparable, and that each measure does not require separate implementation. However, this excludes newer representation learning approaches, or any other methods not implemented in the toolkit. We further explore this choice in the discussion. We also separately perform the experiment on one representation learning approach, OPA2Vec [33], which is compared with results derived from SML measures.

Since there are distinctions between pairwise methods that may or may not admit information content measures, and groupwise measures that may or may not admit pairwise similarity measures, we had to identify the full set of combinations of available methods. In particular, we evaluated combinations of every indirect groupwise measure and every pairwise measure, as well as an additional combination of each IC-using pairwise measure with each IC measure. This led to 300 total experimental settings. We encoded these configurations into a set of XML files that can be used as parameters for the Semantic Measures Toolkit.

**Fig. 2:**
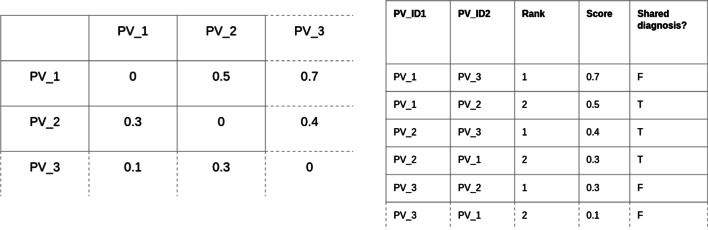
A sample of an example output of the semantic similarity process. On the left is a semantic similarity matrix, in which every phenotype profile associated with a patient visit has been compared with every other one. The result is a matrix of similarity values. To evaluate the matrices, we then convert these into a ranked list of similarity values for each patient visit, which also includes whether or not the two patient visits being compared share a primary diagnosis. The latter structure is used to create our evaluation scores (e.g. AUC). In the experiment described in this article, this process is repeated once for every combination of semantic similarity measure being explored, since each will produce a separate similarity matrix

Each experimental setting, defining a method of measuring similarity between phenotype profiles, produces a pairwise similarity matrix when used to compare every phenotype profile with every other phenotype profile. Subsequently, each of these was was transformed into a set of vectors that associated the semantic similarity score for each pair of admissions with the outcome: whether those admissions shared a primary coded diagnosis. This process is described in Fig. 2.

We used the association between pairwise admission scores and shared diagnosis to evaluate how predictive those scores were of shared primary admission diagnosis, indicated by the associated ICD-9 codes. We evaluated performance using Area Under the receiver operating characteristic Curve (AUC), Mean Reciprocal Rank (MRR), and Top Ten Accuracy (A@10: the percentage of patients for whom the correct diagnosis was in the top ten most similar entities). One previously identified limitation of analyses that compared patient phenotype profiles for prediction of shared diagnosis was that no correct diagnosis can be found for an admission if there is no other admission in the set with a matching primary diagnosis [34]. To identify whether, and to what extent, a lack of matching diagnoses for any admissions in our sample negatively affected scores, we also evaluated a modified MRR metric that removed values of MRR that were 0, since values of 0 indicate there were no admissions with a matching primary diagnosis (and therefore the correct label did not appear at all in the ranking). We also calculated parametric .95 confidence intervals for AUC and A@10 measures. Correlation between evaluation metrics was calculated using the Pearson method. Using these evaluative measures, we interpret the results and compare different methods and classes of methods with each other, and provide discussion and analysis surrounding potential causes for difference.

## Results

We created phenotype profiles for 1000 admissions from MIMIC-III, by identifying HPO terms in their clinical text narrative. We then created semantic similarity matrices for these phenotype profiles using each available combination of pairwise, groupwise, and information content measure. A break-down of different SS and IC method categories is given in Table 1. We did not use two methods listed in the SML documentation. The first was the Tversky information content measure [35], which could not be found in the SML implementation. The second was the Schlicker 2006 measure [36], which requires an additional hyperparameter, and was excluded on that basis, because our experimental design does not allow for hyperparameter optimisation (and this would require further data splitting). This led to a loss of 60 measure combinations, and we therefore obtained results for 300 measure combinations. Using these similarity matrices, we evaluated how predictive the semantic similarity score was of shared primary diagnosis.

**Table 1:** Breakdown of the different categories of semantic similarity measures available in the SML Toolkit

Category	Type of method	Count	Used
Pairwise similarity	Structural	7	7
	Information content	10	8
Groupwise similarity	Direct	19	19
	Indirect (Pairwise required)	5	5
	Indirect (IC only required)	1	1
Information content	Structural	5	5
	Corpus	1	1
Total		48	46

**Fig. 3:**
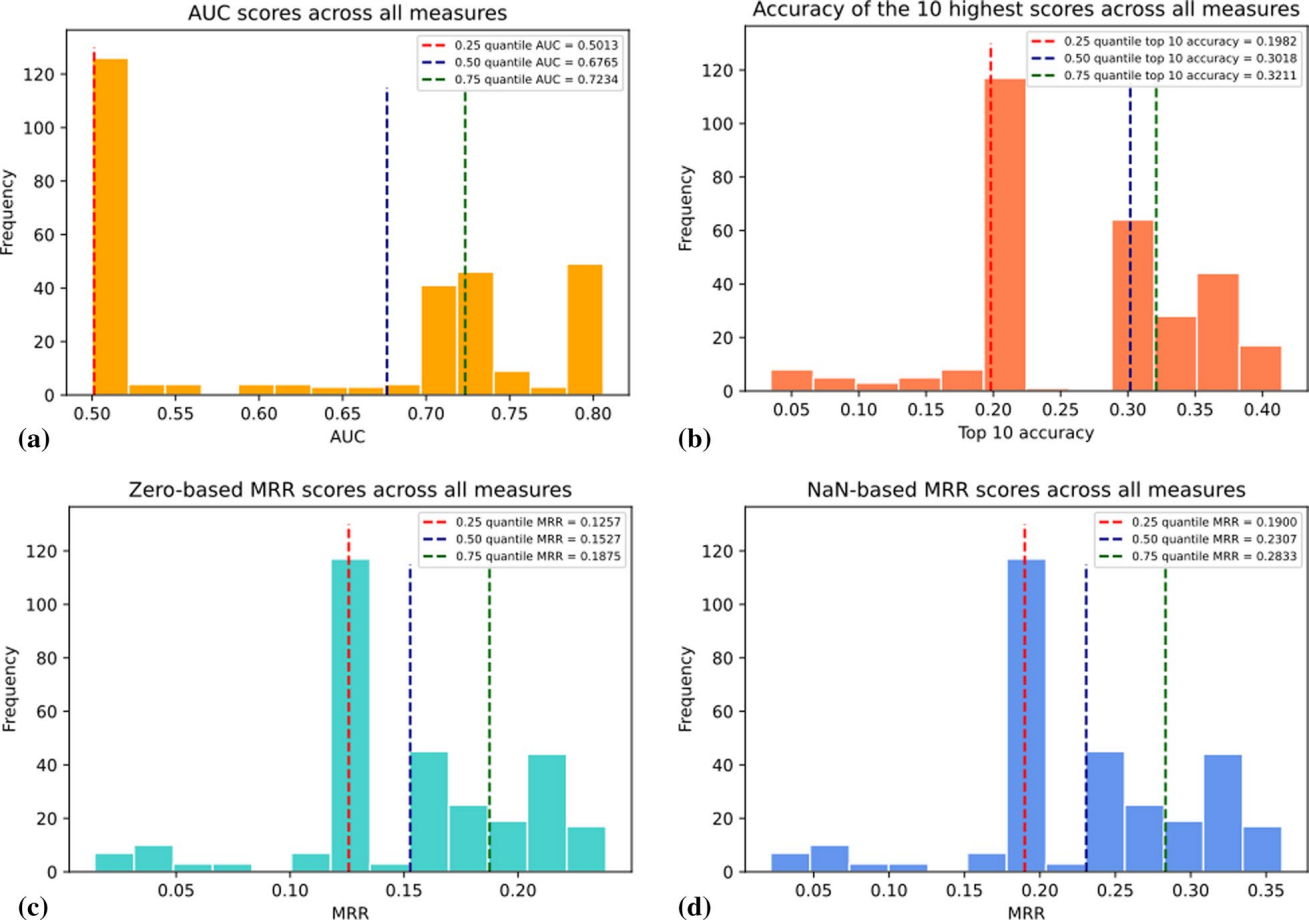
Distribution of scores for all measure combinations evaluated, using different performance measures. The distribution of MRR-0 and MRR-NA are the same, since these scores have a static relationship

Figure 3 shows the distribution of scores for each evaluation metric. In the case of AUC, shown in Fig. 3a, a majority of algorithms fall between 0.5 and 0.6, indicating performance approaching that of a random classifier, and thus that there is no signal in these methods. Interestingly, all other metrics show a median grouping of scores above what would be expected for a completely random classifier, as seen in Fig. 3b–d. This indicates measures that performed poorly by AUC, may nevertheless produce at least one highly ranked correct match, despite overall scoring leading to very poor performance. Correct matches appearing at high ranks by random chance may contribute to baseline performance for the ‘first correct answer’ measures, though in all cases the appearance of scores that fall far below the primary cluster indicates against this.

Nevertheless, ranking of measures correlated mostly strongly between evaluation measures, which we characterised using Pearson correlation. Between AUC and A@10 scores this was 0.68 (*p* value < 2.2e–16), while between AUC and MRR it was 0.6 (*p* value < 2.2e–16), and MRR with A@10 was 0.97 (*p* value < 2.2e–16). Despite these correlations, there are significant exceptions, particularly at the top end of the scores. Table 2, which lists the top three settings for each evaluation measure, confirms this. The GIC (Graph Information Content) measure [37], which admits an information content measure, accounts for all three top measures by A@10, and the top two measures by MRR. The particular information content measure makes minimal contribution to differences in performance here, with all settings being nearly identical by all evaluation measures. However, the AUC of settings involving GIC are substantially lower, at an average of 0.67, than the best performing setting by AUC, of 0.81. The two GIC settings that lead the MRR rankings also have AUC values much lower than the Bader method that appears in third, and which otherwise had identical metrics, excepting a slightly reduced A@10 value. Inversely, however, the best performing measures by AUC suffer from reduced values of the other metrics, which can lead to substantially different results in a practical sense; for example, the best performing measure by A@10 has a matching patient diagnosis in the top ten in 8% more cases than does the best setting by AUC.

**Table 2:** Top algorithms by AUC, MRR, and A@10

By	Method	AUC	MRR-0	A@10
AUC	AVG (GW) + Resnik (PW) + Resnik (IC)	0.81 (0.79–0.83)	0.19	0.34 (0.31–0.37)
	AVG (GW) + Jaccard (PW) + Zhou (IC)	0.8 (0.78–0.82)	0.19	0.32 (0.29–0.35)
	AVG (GW) + NODE_SIM (PW) + Zhou (IC)	0.8 (0.78–0.82)	0.18	0.31 (0.28–0.34)
MRR	GIC (DGW) + Zhou (IC)	0.68 (0.65–0.71)	0.24	0.41 (0.38–0.44)
	GIC (DGW) + Seco (IC)	0.68 (0.65–0.71)	0.24	0.41 (0.38–0.44)
	Bader (DGW)	0.77 (0.74–0.8)	0.24	0.4 (0.37–0.43)
A@10	GIC (DGW) + Resnik (IC)	0.68 (0.65–0.71)	0.24	0.42 (0.39–0.45)
	GIC (DGW) + Sanchez (IC)	0.67 (0.64–0.7)	0.24	0.42 (0.39–0.45)
	GIC (DGW) + Min (IC)	0.67 (0.64–0.7)	0.23	0.42 (0.39–0.45)

Since MRR-NA and MRR-0 are statically dependent, the top algorithms for both are equivalent, and so only ‘MRR-0’ is listed here

All three best performing methods by AUC used the AVG method of indirect groupwise comparison, which averages the result of comparisons made with its associated pairwise method. This could reflect the greater ability of the average method to encode information from large sets of annotation profiles. This is confirmed by the average metric values shown in Table 3 where the best measure by AUC was the AVG, although MRR and A@10 found greater average values through direct measures, which is also in accord with the top-scoring methods shown in Table 2.

**Table 3:** Average performance per groupwise measure

Groupwise method	AUC	MRR-NA	MRR-0	A@10
MAX	0.52	0.19	0.13	0.22
MIN	0.51	0.18	0.12	0.19
BMA	0.68	0.29	0.19	0.33
BMM	0.68	0.22	0.14	0.28
AVG	**0**.**76**	0.24	0.16	0.28
Direct	0.71	**0**.**31**	**0**.**20**	**0**.**36**

Bold indicates the emboldened figure in each column describes the greatest score for the relevant evaluation metric

Table 4 shows average scores for each information content measures, compared with the average scores for methods that did not admit IC methods. This shows that non-IC methods performed better on average by all methods, and with clear separation in all cases. Despite this, we can see another example of deviation from average trends at the top end of performance by noting that the best-performing methods by all three metrics used information content measures.

**Table 4:** Average performance of information content measures versus those that did not use information content

IC method	AUC	MRR-NA	MRR-0	A@10
Resnik	0.62	0.23	0.15	0.26
Zhou	0.62	0.22	0.14	0.25
Seco	0.62	0.22	0.14	0.25
Sanchez	0.63	0.22	0.14	0.25
Max	0.63	0.22	0.14	0.25
Min	0.63	0.22	0.15	0.26
Non-IC	**0**.**67**	**0**.**27**	**0**.**18**	**0**.**32**

Bolded entries in each result column indicate the best-performing method by that measure

We also tested one example of an embedding based approach, OPA2Vec. The AUC was 0.67 (0.64–0.7), the MRR-0 was 0.21, and the A@10 was 0.37 (0.34–0.4). This places it at a medium level of performance with respect to the full set of methods evaluated. The MRR is slightly higher than averages for all groupwise measures, while AUC falls in the middle section, with BMA and BMM, but below AVG and direct measures. It does not appear amongst best performing measures for any metric.

## Discussion

Our experiments revealed a wide range of performances associated with combinations of methods for similarity-based prediction of shared primary diagnosis from text-derived phenotypes, measuring between very poor and good. Despite an overall strong correlation between performance measures, the best-performing methods differed depending on the evaluation metric. This can be accounted for by considering what the metrics measure. MRR measures, on average, the position of the first matching admission for each admission, A@10 measures the percentage of admissions with a matching diagnosis in the top ten most similar admissions, and AUC measures the overall ranking of all matching pairs of admissions, and their position relative to non-matching pairs. Indirect groupwise measures favoured AUC, implying that these produce better overall rankings of shared diagnoses, while direct groupwise measures favoured MRR and A@10, showing that these methods are better for producing highly ranked first matches.

These results provide insight that could, at least in our configuration, be used to inform interface design and interpretation of results in practical applications of semantic similarity to this problem. Methods performing better on MRR and A@10 metrics may be better for direct use by humans, since these are more likely to produce highly ranked correct matches. On the other hand, hybrid approaches that make secondary use of for computation of diagnosis may benefit from measures that perform better by AUC, since aggregated information from multiple matches may be synthesised for improved prediction. Moreover, the AUC measure is derived from similarity score rankings that are calculated globally for all admission pairs, rather than per-admission (as A@10 and MRR are). This means that AUC considers similarity score rankings in global context. As such, it takes into account that while incorrect pairs may be highly ranked in the context of a single admission, the overall ranking of the pair may be appropriately low. This helps to measure the negative predictive power of the approach through its ability to express a low score, where a good match cannot be found. Use of this information in a practical setting would require further development, however, such as the addition of a likelihood ratio or percentage based on a suggestion’s global ranking. However, since these observations could be a feature peculiar to the dataset, or to particular experimental conditions (such as text-derived profiles), additional work should explore other datasets to confirm.

By AUC, settings involving the AVG indirect groupwise measure performed best, while the other two metrics favoured GIC. These methods are in fact relatively similar, with GIC being defined by the average information content of the intersection of terms in the considered sets [37], and AVG being defined by the average result of pairwise comparisons between all terms in the two sets. The major difference between these methods is that GIC considers only exact term matches between the two sets of terms, while AVG admits pairwise methods that measure similarity between individual terms, such as through their most informative common ancestor, and not requiring an exact match (which is the case for the best performing case, using Resnik). This could account for the difference in performance measured by different metrics, as admission pairs with many exact matches would be very highly ranked, while admissions with similar, but not exactly matching, phenotypes, will be lowly ranked in favour of pairs with exactly matching but irrelevant phenotypes (for example, those which are common in the corpus, such as ‘pain’). As such, GIC may produce highly ranked correct answers from pairs where there are an abundance of exactly matching phenotypes, but suffer in cases with fewer exact matches, leading to a lower AUC score. This is an interesting result, that highlights the respective benefits of alternative methodological choices. It is possible that further investigation could be undertaken to identify whether a synthesis of exact and similarity-based methods of semantic similarity could be used to create an approach that combines their virtues. Perhaps, making use of both a local ranking and global ranking, derived from different similarity methods, could lead to a superior method.

The finding that AVG was the best performing indirect groupwise measure by AUC conflicts somewhat with findings for applications to other problems, particularly in relation to genetics and variant prediction [5].

In the case that text-derived phenotype profiles, produced using concept recognition on clinical narrative, contain erroneous annotations of concepts from irrelevant or incorrectly identified mentions of biomedical concepts, information content measures may aid in down-weighting these uninformative associations. For annotation frequency methods, this is because more frequently appearing annotations are down-weighted proportional to the frequency of their appearance. For example, if “allergies” is annotated in every note as a field name, whether an admission has any relevant allergies or not. Term specificity measures evaluate how specific a term is in the ontology used for comparison using its subsumptive hierarchy, which may benefit from a relationship between term generality and likelihood of an uninformative and non-distinguishing mention. For example, the general term “allergies” is more likely to appear in a note erroneously, than a very specific allergy, such as ’allergic conjunctivitis.’ Despite average values for performance being greater for non-IC measures, the best performing measures overwhelmingly involved IC measures, which is in accord with other findings and applications. The average performance of all information content measures was almost equivalent, across annotation-frequency and term-specificity approaches, showing that, in our setting, there is no overall advantage to either method. Meanwhile, overall top methods showed that the annotation-frequency method, Resnik, performed slightly better than term-specificity methods when measuring via AUC and A@10, while best performing methods by MRR used term-specificity (and included one non-IC method).

The significance of the difference between MRR-NA and MRR-0 values is that the MRR-NA value shows us what the MRR value would be if every patient had a match. That these values are much greater, implies that the MRR values are being negative impacted by admissions without a matching admission (one that shared a primary diagnosis). This, in turn, implies that a larger sample size of patients may improve the MRR-0 value close to the MRR-NA value, since there would be fewer unmatched admissions.

We also tested the setting that was used in our previous work, that is the combination of Resnik pairwise, BMA, and Resnik IC [34]. In this experiment, this yielded an AUC of 0.73 (0.7–0.76), and an MRR-0 of 0.23, lower than the previously reported results of 0.77 and 0.42, respectively. We believe this to be caused by patient selection: in the previous experiment, admissions were limited to those with a primary diagnosis that contained a direct mapping in the disease ontology (DO), to facilitate exploration of differential diagnosis with DO disease profiles. As such, we can conclude that the results reported in that paper are optimistic with respect to the full set of diseases described in MIMIC. In addition, these results show that improved performance may have been obtained in this experiment by using AVG as the indirect groupwise measure, instead of BMA.

### Future work and limitations

Since our work only tests one outcome, one ontology, and one dataset, the amount to which the results and interpretation can be extrapolated is limited. However, our work also also provides a framework for more easily evaluating additional experimental configurations and problem applications. It would be possible to introduce different methods of producing text-derived phenotype profiles, curating those profiles, or evaluating prediction of different outcomes, as well as using different ontologies and datasets. There are, however, particular challenges to providing benchmarkable tasks in the area of healthcare applications, since datasets are typically private for ethical reasons. However, other datasets could be tested locally using modifications to our pipeline, and published for comparison. Other datasets, such as OMIM [38] or those provided using the PhenoPackets (https://github.com/phenopackets) schema, could be used to evaluate performance on structured datasets. While our investigation compares ability to classify primary diagnosis, which we use as an indicator of success in measuring true semantic similarity, it does not go further to evaluate grouping on additional factors our outcomes. These tasks require further investigation and development of tailored methods. We hope that the experimental platform described in this work will form the basis for evaluation studies, in a manner similar to those previously described for tasks involving genes and gene products [5].

One such area that the experimental platform could be turned towards, is the prediction of patient diagnosis through comparison with disease profiles, rather than with other patients. In our previous work, we identified that prediction of patient diagnosis from text-derived phenotypes was best when using SS to compare patients to disease profiles mined from literature, and extended with in-context training from patient profiles [34]. We plan to follow this study up with another, comparing patients with different sizes and derivations of disease profile. We do not expect the results of this analysis to necessarily recapitulate the results described in this article, due to the asymmetry of entity types being compared, along with differences in annotation size, source, and composition.

Extension of this work should also consist in its application to different clinical settings. MIMIC-III describes admissions to a critical care setting, which holds particular biases with respect to common diseases and treatment, as well as towards the particular hospital from which the data is derived. As such, results and even best-performing methods may vary depending on many related factors, such as geographic location, jurisdiction, department, or clinical focus.

While this work tests all available methods implemented in the semantic measures library, there are also many semantic similarity approaches implemented as R packages [39], web-based tools [40], and standalone software [41].

There are also newer classes of methods, such as feature learning approaches, that can be used to calculate semantic similarity. However, while SML provides a common interface and abstraction for its methods, these novel approaches are presently presented with entirely bespoke implementations, which may not have been previously applied to the task of annotated entity comparison. While we tested one example of an embedding approach, OPA2Vec, which performed comparably to other methods, its requirement of a completely unique setup and implementation process meant that it was not possible to integrate this with the overall experimental pipeline. This presents a challenge for feasible implementation. Further challenges are presented by other methods, particularly those that make use of machine learning, that require additional training and adaptation to be applied to this problem. For example, Alshahrani discusses a feature learning method that requires training, and has only been implemented for the task of relation prediction [42]. In other cases, models are described, but implementation is not provided. In addition to introducing additional complexity, use of such methods would also necessitate changes to the experimental design, and impact direct comparability with other methods. Further complexity arises in the case of any representation learning approaches, in that there is a distinction between the process of learning representations themselves, and the comparison of those . However, these are sizeable problems in their own right, and are therefore considered outside the context of this work. As an area for future work in benchmarking semantic similarity methods, work should be performed comparing representation approaches with each other, using a range of different methods of calculating similarity between representations, and rule-based approaches. Furthermore, work could be performed in providing guidelines surrounding implementation and reporting of semantic similarity methods, in the same way that MIRO provides guidelines for new ontology reporting [43]. The development of additional shared tasks may also aid in improvement of reporting and gaining comparable results using these methods.

Another limitation of our restriction to measures implemented in SML is that we were unable to measure the amount of time individual methods took to run. This is because in our experimental design leveraged SML’s batch processing feature to perform many calculations at once. In a future experiment, measures could each be run separately. However, theoretical estimates on time consumption can be derived using time complexity reports given in literature presenting individual methods.

## Conclusion

We have presented the development of a platform for evaluating semantic similarity methods for tasks using patient phenotype profiles. We used this implementation to evaluate a large number of settings for the task of predicting shared primary diagnosis from uncurated text-derived phenotype profiles. We interpreted a large range of results by multiple measures, and identified methods that performed more optimally. These results, along with the platform, help to provide a basis for systematically identifying and evaluating methods for practical clinical tasks using semantic similarity methods.

## Data Availability

The framework developed to run this experiment is freely available from https://github.com/reality/mimpred. The annotated MIMIC dataset is not made publicly available, because researchers are required to meet ethical conditions to access MIMIC-derived datasets. However, this can be individually applied for and downloaded separately for use with our software. MIMIC-III can be obtained via https://mimic.physionet.org/, and further instructions are given on the above GitHub repository. The corresponding author can be contacted via email for direct access to the dataset.

## References

[1] Pereira L, Rijo R, Silva C, Martinho R (2015). Text mining applied to electronic medical records: a literature review. Int J E-Health Med Commun (IJEHMC).

[2] Dalianis H (2018). Clinical text mining.

[3] Hoehndorf R, Schofield PN, Gkoutos GV (2015). The role of ontologies in biological and biomedical research: a functional perspective. Br Bioinform.

[4] Gan M, Dou X, Jiang R (2013). From ontology to semantic similarity: calculation of ontology-based semantic similarity. Sci World J.

[5] Pesquita C, Faria D, Falcão AO, Lord P, Couto FM (2009). Semantic similarity in biomedical ontologies. PLoS Comput Biol.

[6] Zhang S-B, Tang Q-R (2016). Protein–protein interaction inference based on semantic similarity of Gene Ontology terms. J Theor Biol.

[7] Schlicker A, Lengauer T, Albrecht M (2010). Improving disease gene prioritization using the semantic similarity of Gene Ontology terms. Bioinformatics.

[8] Hoehndorf R, Schofield PN, Gkoutos GV (2011). PhenomeNET: a whole-phenome approach to disease gene discovery. Nucleic Acids Res.

[9] Köhler S, Schulz MH, Krawitz P, Bauer S, Dölken S, Ott CE, Mundlos C, Horn D, Mundlos S, Robinson PN (2009). Clinical diagnostics in human genetics with semantic similarity searches in ontologies. Am J Hum Genet.

[10] Hoehndorf R, Schofield PN, Gkoutos GV (2015). Analysis of the human diseasome using phenotype similarity between common, genetic, and infectious diseases. Sci Rep.

[11] Liu C, Peres Kury FS, Li Z, Ta C, Wang K, Weng C (2019). Doc2Hpo: a web application for efficient and accurate HPO concept curation. Nucleic Acids Res.

[12] Slater LT, Karwath A, Williams JA, Russell S, Makepeace S, Carberry A, Hoehndorf R, Gkoutos GV (2021). Towards similarity-based differential diagnostics for common diseases. bioRxiv.

[13] Pesquita C, Pessoa D, Faria D, Couto F (2009). CESSM: collaborative evaluation of semantic similarity measures. JB2009: Challenges Bioinform.

[14] Pakhomov S, McInnes B, Adam T, Liu Y, Pedersen T, Melton GB. Semantic Similarity and Relatedness between clinical terms: an experimental study. In: AMIA: annual symposium proceedings. AMIA symposium, vol. 2010;2010. pp. 572–6. PMC3041430 21347043

[15] Lee W-N, Shah N, Sundlass K, Musen M (2008). Comparison of ontology-based semantic-similarity measures. AMIA Ann Symp Proc.

[16] Cornet R, de Keizer N (2008). Forty years of SNOMED: a literature review. BMC Med Inform Decis Mak.

[17] Köhler S, Doelken SC, Mungall CJ, Bauer S, Firth HV, Bailleul-Forestier I, Black GCM, Brown DL, Brudno M, Campbell J, FitzPatrick DR, Eppig JT, Jackson AP, Freson K, Girdea M, Helbig I, Hurst JA, Jähn J, Jackson LG, Kelly AM, Ledbetter DH, Mansour S, Martin CL, Moss C, Mumford A, Ouwehand WH, Park S-M, Riggs ER, Scott RH, Sisodiya S, Vooren SV, Wapner RJ, Wilkie AOM, Wright CF, Vulto-van Silfhout AT, de Leeuw N, de Vries BBA, Washingthon NL, Smith CL, Westerfield M, Schofield P, Ruef BJ, Gkoutos GV, Haendel M, Smedley D, Lewis SE, Robinson PN. The Human Phenotype Ontology project: Linking molecular biology and disease through phenotype data. Nucleic Acids Res 42(Database issue), 2014;966–974. 10.1093/nar/gkt1026. 10.1093/nar/gkt1026 PMC3965098 24217912

[18] Slater LT, Williams JA, Karwath A, Fanning H, Ball S, Schofield PN, Hoehndorf R, Gkoutos GV (2021). Multi-faceted semantic clustering with text-derived phenotypes. Comput Biol Med.

[19] Kulmanov M, Hoehndorf R (2017). Evaluating the effect of annotation size on measures of semantic similarity. J Biomed Seman.

[20] Shefchek KA, Harris NL, Gargano M, Matentzoglu N, Unni D, Brush M, Keith D, Conlin T, Vasilevsky N, Zhang XA, Balhoff JP, Babb L, Bello SM, Blau H, Bradford Y, Carbon S, Carmody L, Chan LE, Cipriani V, Cuzick A, Rocca MD, Dunn N, Essaid S, Fey P, Grove C, Gourdine J-P, Hamosh A, Harris M, Helbig I, Hoatlin M, Joachimiak M, Jupp S, Lett KB, Lewis SE, McNamara C, Pendlington ZM, Pilgrim C, Putman T, Ravanmehr V, Reese J, Riggs E, Robb S, Roncaglia P, Seager J, Segerdell E, Similuk M, Storm AL, Thaxon C, Thessen A, Jacobsen JOB, McMurry JA, Groza T, Köhler S, Smedley D, Robinson PN, Mungall CJ, Haendel MA, Munoz-Torres MC, Osumi-Sutherland D (2020). The Monarch Initiative in 2019: an integrative data and analytic platform connecting phenotypes to genotypes across species. Nucleic Acids Res.

[21] Smedley D, Oellrich A, Köhler S, Ruef B, Sanger Mouse Genetics Project, Westerfield M, Robinson P, Lewis S, Mungall C. PhenoDigm: analyzing curated annotations to associate animal models with human diseases. Database J Biol Databases Curation. 2013;2013:025. 10.1093/database/bat025. 10.1093/database/bat025 PMC3649640 23660285

[22] Robinson PN, Kühler S, Oellrich A, Sanger Mouse Genetics Project, Wang K, Mungall CJ, Lewis SE, Washington N, Bauer S, Seelow D, Krawitz P, Gilissen C, Haendel M, Smedley D. Improved exome prioritization of disease genes through cross-species phenotype comparison. Genome Res. 2014;24(2):340–348. 10.1101/gr.160325.113. 10.1101/gr.160325.113 PMC3912424 24162188

[23] Meng L, Huang R, Gu J (2013). A review of semantic similarity measures in wordnet. Int J Hybrid Inf Technol.

[24] Rada R, Mili H, Bicknell E, Blettner M (1989). Development and application of a metric on semantic nets. IEEE Trans Syst Man Cybern.

[25] Zhou Z, Wang Y, Gu J. A new model of information content for semantic similarity in WordNet. In: 2008 Second international conference on future generation communication and networking symposia, vol. 3. IEEE, Hinan, China; 2008. pp. 85–9.

[26] Resnik P. Using information content to evaluate semantic similarity in a taxonomy. arXiv:cmp-lg/9511007. 1995.

[27] Perozzi B, Al-Rfou R, Skiena S. Proceedings of the 20th ACM SIGKDD international conference on knowledge discovery and data mining. Deepwalk: online learning of social representations. 2014. pp. 701–10.

[28] Mikolov T, Sutskever I, Chen K, Corrado GS, Dean J. Distributed representations of words and phrases and their compositionality. In: Burges, C.J.C., Bottou, L., Welling, M., Ghahramani, Z., Weinberger, K.Q. (eds.) Advances in neural information processing systems, vol. 26. Curran Associates, Inc., Lake Tahoe; 2013. https://proceedings.neurips.cc/paper/2013/file/9aa42b31882ec039965f3c4923ce901b-Paper.pdf.

[29] Grover A, Leskovec J. Node2vec: scalable feature learning for networks. In: KDD: proceedings. International conference on knowledge discovery & data mining, vol. 2016; 2016. pp. 855–64. 10.1145/2939672.2939754. 10.1145/2939672.2939754 PMC5108654 27853626

[30] Johnson AEW, Pollard TJ, Shen L, Lehman L.-w.H, Feng M, Ghassemi M, Moody B, Szolovits P, Celi LA, Mark RG (2016). MIMIC-III, a freely accessible critical care database. Sci Data.

[31] Slater LT, Bradlow W, Hoehndorf R, Motti DF, Ball S, Gkoutos GV (2020). Komenti: a semantic text mining framework. bioRxiv.

[32] Harispe S, Ranwez S, Janaqi S, Montmain J (2014). The semantic measures library and toolkit: fast computation of semantic similarity and relatedness using biomedical ontologies. Bioinformatics.

[33] Smaili FZ, Gao X, Hoehndorf R. OPA2Vec: combining formal and informal content of biomedical ontologies to improve similarity-based prediction. arXiv:1804.10922 [cs]. 2018. 10.1093/bioinformatics/bty933 30407490

[34] Slater LT, Karwath A, Williams JA, Russell S, Makepeace S, Carberry A, Hoehndorf R, Gkoutos GV (2021). Towards similarity-based differential diagnostics for common diseases. Comput Biol Med.

[35] Blanchard E, Harzallah M, Kuntz P (2008). A generic framework for comparing semantic similarities on a subsumption hierarchy. ECAI.

[36] Schlicker A, Domingues FS, Rahnenführer J, Lengauer T (2006). A new measure for functional similarity of gene products based on Gene Ontology. BMC Bioinform.

[37] Pesquita C, Faria D, Bastos H, Falco A, Couto F. Evaluating GO-based semantic similarity measures. In: Proceedings of 10th annual bio-ontologies meeting. 2007.

[38] Hamosh A, Scott AF, Amberger JS, Bocchini CA, McKusick VA (2005). Online Mendelian Inheritance in Man (OMIM), a knowledgebase of human genes and genetic disorders. Nucleic Acids Res.

[39] Ovaska K, Laakso M, Hautaniemi S (2008). Fast Gene Ontology based clustering for microarray experiments. BioData Min.

[40] Couto FM, Silva MJ, Coutinho PM. Implementation of a functional semantic similarity measure between gene-products. 2003.

[41] Le D-H (2020). UFO: a tool for unifying biomedical ontology-based semantic similarity calculation, enrichment analysis and visualization. PLoS ONE.

[42] Alshahrani M, Khan MA, Maddouri O, Kinjo AR, Queralt-Rosinach N, Hoehndorf R (2017). Neuro-symbolic representation learning on biological knowledge graphs. Bioinformatics.

[43] Matentzoglu N, Malone J, Mungall C, Stevens R (2018). MIRO: guidelines for minimum information for the reporting of an ontology. J Biomed Seman.

